# Self-categorization as a basis of behavioural mimicry: Experiments in The Hive

**DOI:** 10.1371/journal.pone.0241227

**Published:** 2020-10-30

**Authors:** Fergus G. Neville, John Drury, Stephen D. Reicher, Sanjeedah Choudhury, Clifford Stott, Roger Ball, Daniel C. Richardson

**Affiliations:** 1 School of Management, University of St Andrews, St Andrews, United Kingdom; 2 School of Psychology, University of Sussex, Brighton, United Kingdom; 3 School of Psychology & Neuroscience, University of St Andrews, St Andrews, United Kingdom; 4 School of Psychology, Keele University, Keele, United Kingdom; 5 Division of Psychology and Language Sciences, University College London, London, United Kingdom; University of California Los Angeles, UNITED STATES

## Abstract

**Introduction:**

Do we always do what others do, and, if not, when and under what conditions do we do so? In this paper we test the hypothesis that mimicry is moderated by the mere knowledge of whether the source is a member of the same social category as ourselves.

**Methods:**

We investigated group influence on mimicry using three tasks on a software platform which interfaces with mobile computing devices to allow the controlled study of collective behaviour in an everyday environment.

**Results:**

Overall, participants (N = 965) were influenced by the movements of confederates (represented as dots on a screen) who belonged to their own category in both purposive and incidental tasks.

**Conclusion:**

Our results are compatible with collective level explanations of social influence premised on shared social identification. This includes both a heuristic of unintended mimicry (the acts of group members are diagnostic of how one should act), and communication of affiliation (based on a desire to make one’s group cohesive). The results are incompatible with traditional ‘contagion’ accounts which suggest mimicry is automatic and inevitable. The results have practical implications for designing behavioural interventions which can harness the power of copying behaviour, for example in emergency evacuations.

## Introduction

When–and why–do we copy the behaviour of others? Such mimicry applies to an extremely wide range of phenomena from the simplest reactions such as scratching and yawning [1,2] to more complex activities such as health related behaviours [3] and from the self-evidently purposive–such as food consumption [4]–to the apparently incidental, such as face touching [5], finger tapping [6], and foot shaking [7].

Mimicry has also been invoked as a key factor in building bonds between individuals [8,9]. Some have gone further and argued that it is the basic building block of all social life [2,10]. The importance of mimicry is evidenced by the number of human sciences that draw upon this concept, including psychology [11], economics [12], and consumer behaviour [13], but also in animal research [14].

However, while there is widespread consensus as to the importance of the phenomenon, there is far less agreement as to the condition(s) under which it occurs. In this paper, we draw on self-categorisation theory (SCT) [15] in order to test the hypothesis that mimicry is moderated by the mere knowledge of whether others are members of the same social category as ourselves.

### The nature and causes of mimicry

Mimicry is a form of indirect social influence where the source is copied by an interaction partner without that source directly seeking to exert influence [16]. It is part of a broader set of copying phenomena, including emulation (which involves copying the results without necessarily using the same exact actions to achieve them) [17] and imitation. Mimicry involves an interaction partner copying the actual behaviour of the source within a short window of time, typically no longer than three to five seconds [16]. Mimicry is usually thought of as distinct from conformity and normative influence, which are associated with an internalisation of beliefs or attitudes rather than the adoption of a fleeting behaviour [18,19]. Other terms are often used to denote influence without conscious intent, notably ‘contagion’ [20]. However, in this paper we avoid the term ‘contagion’ or else use it in quotation marks because of the way it conflates a description of the phenomenon with a (medicalised) explanation and a (negative) evaluation. The metaphor of ‘contagion’ suggests that mimicry, like a virus, spreads by mere proximity alone, regardless of who the carriers are, or the relationships involved.

A number of researchers propose that mimicry is used to communicate about oneself and is used as a tool to affiliate with other individuals [11,21]. When, in interpersonal contexts we copy the emotions, facial expressions or gestures of others, we convey understanding and a sense of togetherness with the other. Accordingly, it has been shown across various studies, that mimicry increases when participants believe that they are visible to others [6,22] and when participants have a desire for affiliation with ingroup members [9,23,24] or to appease outgroup members [25,26]. However, it is possible that mimicry has functions other than communication and affiliation, which would be indicated if it occurred in the absence of a watching audience. An alternative heuristic approach suggests that we copy others because their behaviour is likely to be a good guide to how we should behave ourselves [27,28]. In an emergency evacuation, for example, others’ egress behaviour may be the only information available about appropriate exit routes and will therefore lead to copying. Using a virtual reality experiment in which people had to escape from a room in a museum, Kinateder, Comunale and Warren showed that using a familiar exit in an emergency evacuation is increased if neighbours do the same [29]. Van den Berg and colleagues used computer gaming to show that the more people someone sees leaving in a potential emergency, the more inclined this person is to leave [30]. This is not to say that the use of a mimicry heuristic is always adaptive in terms of its outcomes. Virtual reality studies have shown that participants follow the behaviour of computer-generated agents during fire evacuations even if this delayed their own egress [29,31].

On the other hand, as studies of behaviour in emergencies show, we don’t follow everyone equally [32,33]. There are a range of studies which show that mimicry is more likely when one has some sort of social bond to the other [7,34]. This is true for behavioural ‘contagion’, whether of yawning [35], face touching [36] or key-pressing [37]. It is equally true of emotional ‘contagion’ [21,38–40].

The notion of ‘social bond’, however, is somewhat vague in most of these studies. In some cases, it refers to close interpersonal relationships [35]. In other cases, it refers to a social categorical relationship whereby the source and the observer are part of a common social group [39]. Our interest lies particularly in the latter, not least because it is more relevant to large-scale social instances of mimicry where the source and observers may not be close to each other and may even be strangers. Moreover, drawing on contemporary models of group process–specifically SCT [15,41]–we aim to examine together the two core dimensions of the mimicry process: first, which others do we mimic; second why do we mimic others?

### A self-categorisation analysis of mimicry

According to SCT, and social identity theory from which it developed [42,43], the human self is not a unitary construct but rather a multi-dimensional system made up of personal identities (which define what makes me as an individual distinctive compared to other individuals) and social identities (which define what makes my group distinctive compared to other groups). We have multiple social identities, corresponding to the different groups we belong to, which become salient in different contexts. When any given social identity is salient, it determines how we see others (notably whether they are ingroup or outgroup) and defines the norms, values and beliefs through which we see the world. Insofar as ingroup members share in these understandings we expect to agree with them [44]; we actively seek agreement with them [45]; and, especially on matters of relevance to the group, we see them as a guide to what we should think and do [46,47].

In terms of mimicry, then, the SCT approach is consistent with the heuristic approach of Gigerenzer [28] by specifying that we see others as providing diagnostic behaviour as to what we ourselves should do, but only to the extent that we categorise ourselves with them as fellow ingroup members. One implication of this is that we may mimic complete strangers as much or even more than those we know well under conditions where the former are ingroup and the latter are not. To take a familiar example, at a sports event, I may copy the behaviour of an unknown other who supports the same team as myself while ignoring my sister or friend if they were to support the other team.

To date, self-categorisation researchers have devoted considerable efforts to showing that we are more likely to be influenced by those who are ingroup than those who are outgroup [47,48]. However, the research has generally been focussed on explicit acts of persuasion. With the exception of some qualitative illustrations in the context of crowd events [46] no attention has been paid to the immediate copying of an other’s behaviour in the absence of an explicit attempt by the source to shape the behaviour of the observer. That is the gap which we aim to fill with this paper.

### The present study

We investigated group influence on mimicry using an innovative software platform—The Hive—which integrates with mobile computing devices to allow collective behaviour to be studied in a controlled environment. In The Hive, physically co-present participants move a dot around the screen of their phone or tablet. On a shared central display, they can see their own dot and the dots of all the other participants as well. The display can show images and videos with the dots superimposed, so that participants can take part in an experimental task by moving their dot. The experimenter can also change the colour of the dots or make them invisible to participants. The Hive has been used to investigate, for example, how knowledge of each other’s responses changes individual and collective decisions [49].

In our experiment, participants took part in a number of simple games while divided up into red or blue groups. We employed techniques for inducing psychological group membership analogous to those used in the minimal group paradigm [50]. That is, participants were assigned into groups at random, although led to believe that they had been grouped with people who had made similar choices to them in a personality quiz or in an art preference task. Although in each trial participants were sitting next to each other, they did not know who was in their group; and being anonymous, they were not able to communicate with each other. After the minimal group assignment, we introduced confederate dots into the display. Although they appeared as other participants, these dots behaved in a pre-programmed way according to their colour.

Across three tasks, we investigated whether participants would mimic the behaviour of the confederate dots in their colour group. In the ‘Maze’ task, the red and blue confederates moved to targets on different sides of the screen. In the ‘Rather’ task, we asked participants questions such as ‘would you choose the power of flight or invisibility?’ Participants had to move to the left side or right side of a box to indicate their magnitude of certainty for one choice or another. The blue and red confederates always clustered towards a particular choice. In addition, the red confederates left their dots higher in the box than the blues. Third, in a “Fidget” task, participants were told to wait inside a circle between questions while red confederates moved their dots around slightly and the blues stayed still.

There are four key elements of these tasks which need highlighting. First, the directions and distances travelled by the participants’ dots in relation to the confederate dots of a similar or different colour provides measures of ingroup and outgroup mimicry. Our core prediction is that, on each of the experimental tasks, participants will mimic the ingroup dots more than the outgroup dots.

Second, in some cases mimicry can be seen as purposive in the sense of being linked to the fulfilment of the experimental task (i.e., the ‘maze’ task and the left-right choice on the ‘rather’ task). In other cases mimicry can be seen as incidental in the sense of being irrelevant to the fulfilment of the experimental task (i.e., the height choice on the ‘rather’ task and the ‘fidget’ task). Overall, then, the range of tasks allows us to see if the effects of shared category membership on mimicry will be limited to either purposive or incidental behaviours, or else relevant to both. Our hypothesis is that it will be relevant to both.

Third, other than their own dot, participants have no idea which dot is associated with whom. They don’t know if any given dot represents someone they know well or a complete stranger. All they know is whether the dot represents an ingroup or an outgroup member. It follows that any effects of group relations on mimicry cannot be put down to our personal relations with other group members but merely from the knowledge of our shared group membership with them.

Fourth, participants are also aware that others have no way of knowing which dot is associated with whom. Consequently, the way they move their dot cannot be used to communicate anything about themselves as individuals to others. It flows from this that, any effects of group relations on mimicry, cannot be explained in terms of communicating about oneself or as a means of securing one’s affiliation with other group members.

## Methods

### Participants

The experiment ran as a summer residency in the ‘Who Am I?’ gallery of the Science Museum, London, as part of their ‘Live Science’ programme. Participants were visitors to the museum who agreed to take part in a live study entitled ‘How do you behave in a crowd?’. They were recruited by posters and flyers in the museum, or by experimenters approaching them on the gallery floor. Participants were not compensated for their time, as their motivation was to learn about social science. We sought to run participants in groups of 6–8 with ages of 10 and above. Since this was an opportunity sample with an educational remit, however, we did not want to turn away willing participants. With these constraints, group sizes varied from 4 to 12, with a median of 7 (M = 7.58, SD = 1.88).

Across six weeks of our residency, 1139 people participated in our experiment. For the analysis, we excluded participants under 10 years of age, and participants who did not complete the task by choice or because of technical error (such as their device losing internet connection). This left us with 965 participants to analyse. 55% of participants were female, 35% male and 10% declined to provide their gender, and overall, they had an age range of 10 to 71 (M = 26.33, SD = 13.87).

### Procedure

The experiment ideally began once 6–8 participants had been recruited to take part. Sometimes, if the museum was especially quiet or busy, we ran groups as small as 4 or as large as 12. Participants were told it was a ten-minute experiment investigating ‘how you behave in a crowd’. Participants were given an information sheet and a consent form to sign. If they were under 16 their parent or guardian signed a form as agreed by our ethics committees and the Science Museum. The experiment was reviewed and approved by the UCL Research Ethics Committee (Project ID Number: 3828/003) and the School of Psychology & Neuroscience Ethics Committee at the University of St Andrews (Approval Code: PS12971).

**Fig 1:**
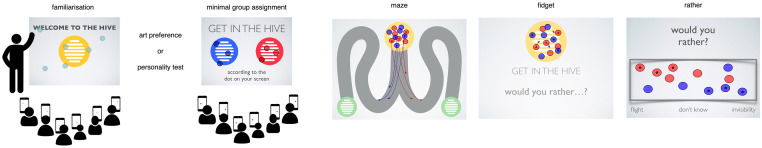
Schematic of experiment set up and minimal group assignment. In each task, confederate dots are shown here with black dots in the middle, though to participants, they were indistinguishable from their own. The ‘rather’ task used images to illustrate ‘flight’ and ‘invisibility’ which have been removed here for copyright reasons.

Participants sat on curved benches surrounding a large LCD display in a cordoned area of the Science Museum. Using their own mobile device, or a tablet that we supplied, participants accessed The Hive website. They entered their age and gender and a unique code for the experimental session. After logging on, their device displayed a dot that could be dragged around. Each participant saw their own dot, and other participants’, moving on the central display (see Fig 1). An experimenter stood by the central display and welcomed the participants once they were all logged on to the Hive. Participants were asked several warm-up questions so that they could familiarise themselves with using The Hive. Participants were assured that all their data was stored anonymously and that they could withdraw and request that their data be removed at any time.

Using one of two minimal group paradigms, participants were next assigned to either a red or a blue group. In the *art* paradigm, participants moved their dot to express which of 12 modern art paintings they preferred. In the *personality* paradigm, they answered the 10 items from the Ten Item Personality Measure (TIPI) [51] using an onscreen Likert scale. In both paradigms, participants’ dots were not visible on the central display, so that they could give their response privately without being influenced by others. Participants were then told that based on their responses, we had categorised them individually into either the red or the blue group, such that they were placed with people who were similar to themselves and in a different group from those who were dissimilar to them. Participants’ dots then changed colour on their devices and the central display, and they were asked to move to different sides of the screen depending on their assigned group.

At this point the experimenter mentioned that ‘other people playing online or elsewhere in the museum may be taking part in our experiment too’. Either 6 or 10 confederate dots then appeared onscreen, with an equal number of reds and blues. The confederate dots’ movements were pre-recorded by the experimenters, but they appeared to the participants as real, live participants indistinguishable from those in the museum. Participants then took part in a series of games and decisions.

#### Maze task

Participants were told to move into a central yellow circle at the top of the screen. The task was to move to one of the green circles in the bottom left or right of the screen, while keeping their dot within one of two dark grey paths. They were told that they could freely choose which green circle to choose. Red and blue confederates were programmed to move in opposite directions to either the left or right circle. The maze task had one trial and participants were given 15 seconds to complete the task.

#### Rather task

Participants were asked whether they would rather have the superpower of flight or invisibility, have love or money, be a dragon or own a pet dragon, and be covered in fur or scales. To give their answer they moved from the top, centre of the screen into a response box with one option on the left, one on the right, and ‘don’t know’ in the middle. Red confederate dots tended to cluster higher in the response box than the blue, and the two colours tended to cluster towards different options.

#### Fidget

Participants were asked to move to a central yellow circle on four occasions to await the next ‘Would you rather?’ question. In the ten seconds that they waited, the red confederates fidgeted within the circle, moving around slightly from side to side. The blue confederate dots remained still. This task was not counterbalanced.

After these experimental tasks, participants took part in a brief selection of other activities in The Hive that varied during the course of the six-week residency. These tasks were investigating unrelated hypotheses, and so are not reported here. Importantly, they all occurred after the experimental task, and so could not influence the results. At the end of the experiment, participants were debriefed and given a short talk about our hypotheses and related findings in the literature.

### Design

Our key independent variable was the dot *colour* that was randomly assigned to participants. In addition to this, we varied a number of other factors between participants. For each session, we manipulated the *grouping* method, by art preference or personality test. We varied the number of *confederate* dots between 6 and 10. And we varied the *orientation* of the experimental tasks: for half of the sessions, we mirror reversed the motions of the confederate dots, so that they chose different sides on the maze task and inverted their choices on the preference task.

## Results

Overall, participants were influenced by the movements of the confederate dots who were the same colour as their own. The exception was the ‘Rather’ task: when we asked participants to express an individual, arbitrary preference, there was no influence from the confederate dots’ movements.

Across our different experiments, we used Bayesian mixed models to quantify the evidence that a participant’s dot colour affected their behaviour. Mixed models are able to account for the effect of individual participants being nested in a particular group, and the Bayesian approach avoids some of the problems associated with null hypothesis testing [52–55]. Each of our Bayesian mixed models used fixed effects for the participants’ dot *colour* (red or blue), the number of *confederates* (6 or 10), the *grouping* manipulation used (TIPI personality index or art preference), the effect of *orientation* (where relevant), and a random effect for the group. We used random slopes and random intercepts. We used R (version 3.4.3) and the *rstanarm* package [56], employing weakly informative priors that were scaled following the standard rstanarm procedure. From 4000 simulations, we generated estimates of the posterior distributions of the model parameter coefficients, which quantify the strength of the evidence that each experimental condition influenced behaviour.

Below we report the estimates of the differences between experimental conditions, using the psycho R package [57]. We report the Maximum Probability of Effect (MPE), which is the probability that the absolute value of the effect has a median greater than zero. In other words, the MPE directly quantifies the probability that the experimental condition had an effect on behaviour. The Bayesian approach favours quantifying the strength of evidence in this way, rather than simply reporting whether or not an (arbitrary) threshold of significance has been passed. Having said that, researchers generally suggest that an MPE of above 90% or 95% can be thought of as ‘strong evidence’ [58]. In addition to these Bayesian analyses, we ran frequentist analysis using more conventional mixed models. These produced a corresponding pattern of results and can be seen in the SI.

### Maze task

We looked at which side of the screen the participants moved to by the end of the trial and coded this as either the blue side (dot side = 0) or the red side (dot side = 1), according to where the confederate dots had moved. In half of the trials, the blue confederates moved to the left, on the other half they moved to the right. Our hypothesis, therefore, was that if the participants had a red dot their dot side score would be more likely to be 1, and a blue dot, 0.

**Fig 2:**
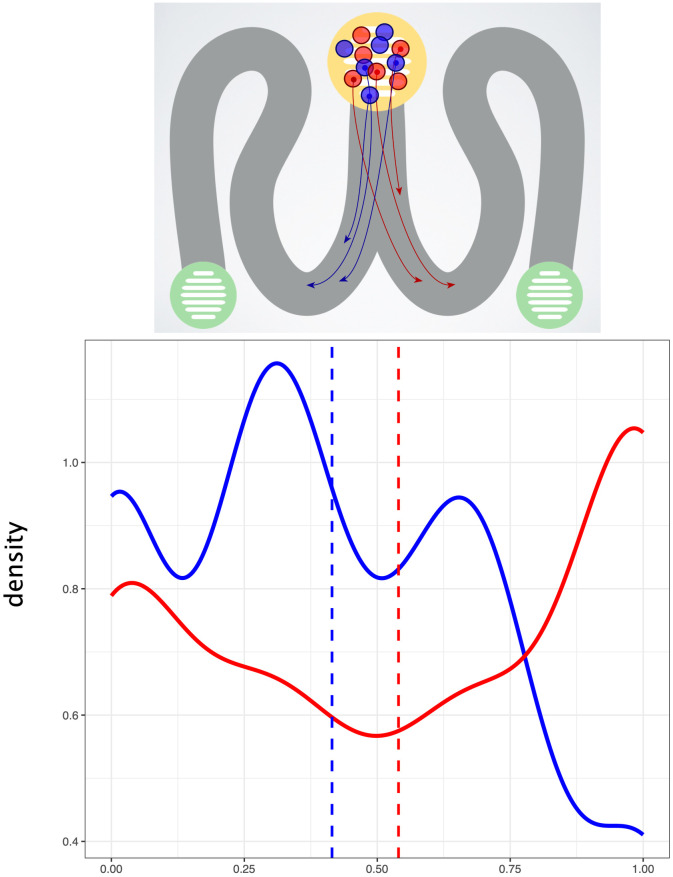
Distribution of participants’ maze choices, split by dot colour. Inset of example trial with confederate dots identified by arrows showing pre-determined movements.

In Fig 2 we averaged the dot side scores for each group of participants, to give a continuous variable. As can be seen, the dot side score was higher for red dots than blue.

Our Bayesian mixed model analysed individual participants’ dot side, nested in their experimental group. Since our dependent variable was binary in this case, we based the model on a binomial distribution. For this experiment, we also included a fixed effect of *orientation* to account for the fact that the direction that the red and blue confederates travelled was reversed for half of the experimental groups. For a fuller description of the Bayesian analysis and frequentist mixed models for the maze task (which come to the same conclusion), please see S1–S3 Tables.

The colour of participants’ dots influenced which side of the maze they chose. The model estimated that red dots had a 57% chance of going to the same side as the red confederates, and the blue dots had a 42% chance (in other words, 58% chose to go to the same side as the blue confederates). The MPE for this effect of colour was 99.99%, suggesting strong evidence for the effect seen in Fig 2.

We also found an effect of the orientation condition (MPE = 99.99%). It seems that, overall, there was a tendency for blue dots to go to the left and red dots to go to the right. The most obvious interpretation of this result is that immediately prior to the maze trial, the participants were asked to move to a side of the screen according to their dot colour, and this was always blue on the left and red on the right. In the maze trial, half of the time blue confederates continued to move to the left, and half the time they moved to the right (and red vice versa). However, there was evidence that participants continued to be influenced by this initial spatial assignment.

Despite this overall bias, we found strong evidence that participants were influenced by the behaviour of their same-colour confederates. MPEs for the other parameters suggested that they did not differ from zero. In other words, there was no evidence that the other two experimental conditions–type of grouping, and number of confederates–had an influence on participants (see SI for all parameter estimates and MPEs).

### ‘Rather’ task

**Fig 3:**
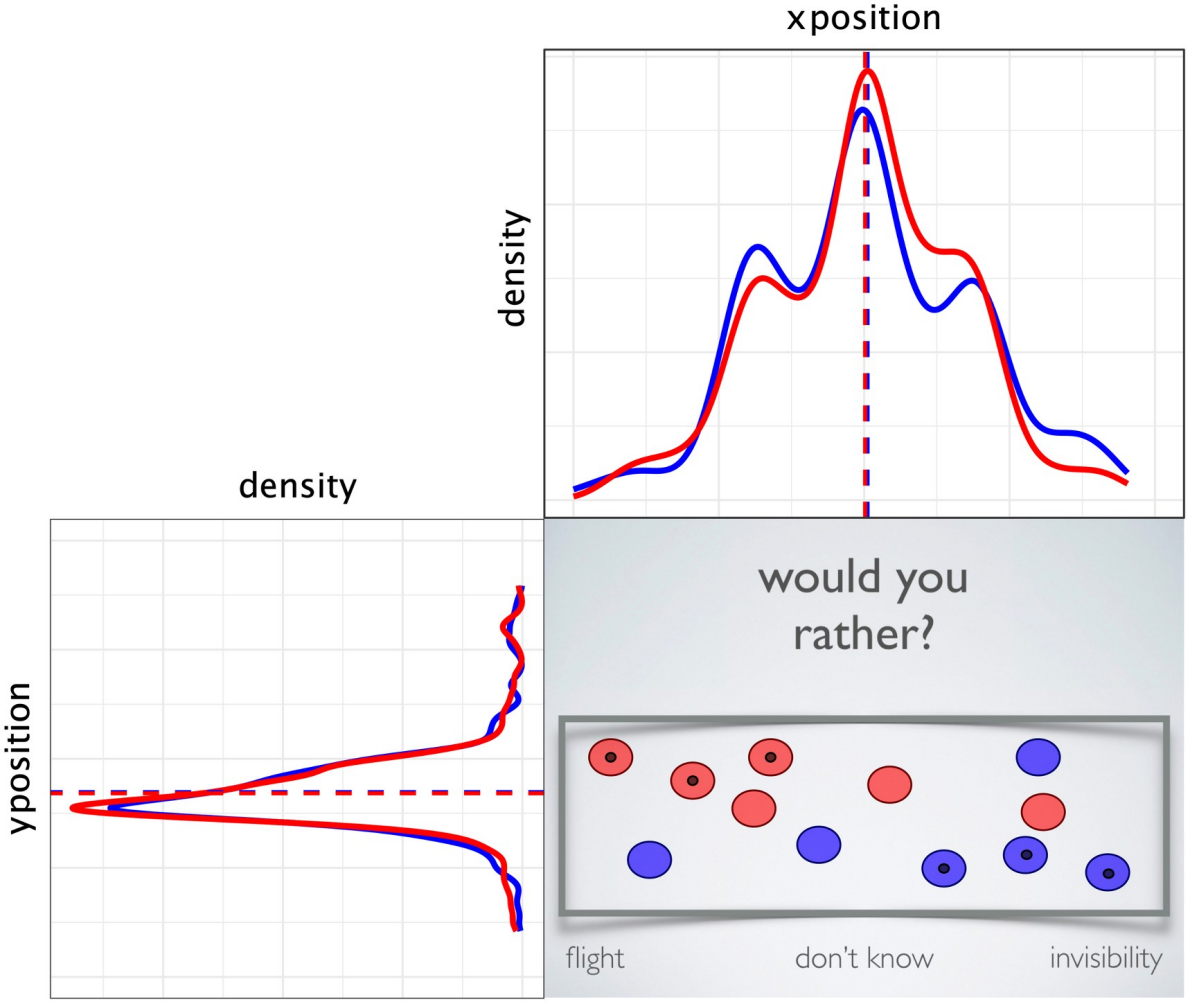
Example of the final stage of a preference trial. Confederate dots, identified with a dark spot, clustered in corners of the response box. Density plots show participants’ final vertical (shown left) and horizontal (top) dot positions. The ‘rather’ task used images to illustrate ‘flight’ and ‘invisibility’ which have been removed here for copyright reasons.

We analysed the participants’ final horizontal and vertical dot positions separately, to see if either were influenced by the confederate dot positions of a particular colour. The confederates clustered in different corners according to their colour on each trial. Data were then mirror reversed according to counterbalancing, so that the red confederate dots clustered in the top left of the response box, and blue confederates on the bottom right (as in the example shown in Fig 3). This allowed us to frame our predictions more simply by saying that if the explicit element of participants’ choice was influenced by their confederate group members, then the horizontal position of the blue dots should be greater than the red. If the arbitrary element of participants’ choice was influenced by their confederate group members, then the red dots should have higher vertical position than the blues.

As can be seen in Fig 3, there appeared to be no influence of participants’ dot colour on their vertical or horizontal position. Our Bayesian mixed models for this analysis had the addition of a random effect for *item*, since there were four different questions. The models estimated that the effect of colour was essentially at chance levels for the vertical position (MPE = 59.9%) and the horizontal position (MPE = 52.6%). For a fuller description of the Bayesian analysis and frequentist mixed models for the rather task (which come to the same conclusions), please see S4–S9 Tables.

### Fidget

**Fig 4:**
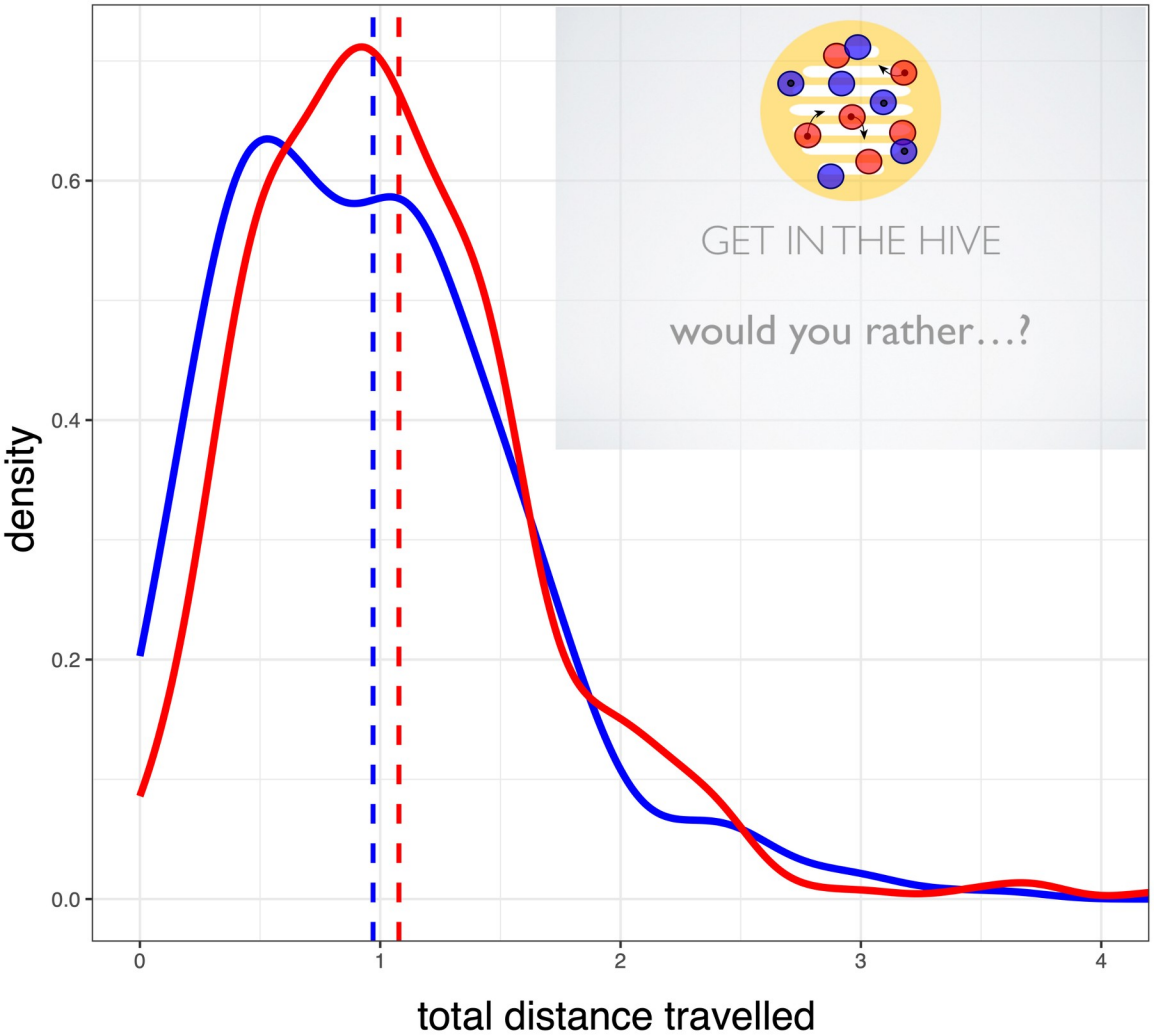
Distribution of the total distance participants travelled while waiting inside the yellow circle, split by dot colour. Inset shows an example trial, with confederate dots marked with a spot.

We calculated the total distance travelled by the red and the blue dots once they had entered The Hive circle, for the ten seconds that they waited for the next question to appear in the ‘Rather’ task. The red confederate dots fidgeted during this time, moving around slightly within the circle, whereas the blue confederate dots did not. We summed the distance travelled by the participants (excluding the confederates) across four such trials (Fig 4). As we hypothesised, this distance was greater for the red dots, who travelled roughly 10% farther than the blue.

Our Bayesian mixed model found strong evidence that colour influenced participant dot movement (MPE = 98%). We also found evidence that grouping manipulation type (personality or art) influenced movement (MPE = 99.99%). Participants of both colours who believed that they had been assigned their dot colour on the basis of the personality test moved their dots more during the pre-‘Rather’ tasks waiting periods. All parameter estimates of our Bayesian mixed models, and the results of a frequentist mixed model showing the same pattern of significance, are reported in the SI. The grouping manipulation did not interact with participants’ dot colours. Regardless of these differing overall levels of restlessness, participants were still influenced by the small fidgeting motions of the dots whose colour they shared. For a fuller description of the Bayesian analysis and frequentist mixed model analysis for fidgeting (which come to the same conclusion), please see S10–S12 Tables.

## Discussion

This paper reported the results from three tasks in which participants used a new interactive paradigm to make behavioural choices within virtual collective environments. Participants each controlled a dot on a shared display and were divided into two colour groups apparently based on their responses to personality questions or art preferences. Pre-programmed confederate dots moved in specific ways which allowed testing of self-categorization and communication explanations for behavioural mimicry in both purposive and incidental tasks.

In the ‘Maze’ task participants were more likely to pick the same direction of travel as their same-coloured confederates, and in the ‘Fidget’ analysis participants travelled further if they observed same-coloured dots (but not different-coloured dots) moving more. However, in the ‘Rather’ task there was no significant difference in final vertical or horizontal position based on the movements of same-coloured dots.

In sum, our predictions are upheld in two of the three tasks, covering both purposive (‘Maze’) and incidental (‘Fidget’) cases. The exception is the ‘Rather’ task where our prediction that group membership would moderate mimicry was not upheld. In hindsight, perhaps this is not altogether surprising. SCT does not propose that we are guided by fellow ingroup members whatever they do. It makes clear that influence will be limited to matters that are of relevance to the group identity.

In the present context, then, the wording of the ‘Rather’ task makes explicit that this is an individual preference rather than a group relevant choice (e.g., “Would *you* rather be a dragon or have a pet dragon?”–emphasis added) and therefore we would not expect group influence to apply. Rather than undermining our overall position, then, this lack of mimicry on the ‘Rather’ task serves to refine our hypotheses by adding an additional dimension: mimicry of any given action will depend both on the source being ingroup and the action not being explicitly irrelevant to group identity. This suggests the importance of addressing issues of identity content alongside issues of source identity in future research on mimicry.

In terms of explaining the source effects on mimicry that we did find for the ‘Maze’ and ‘Fidget’ tasks, these findings further support the argument that such social influence is not passive and automatic [5,21,39,59–63]. People clearly don’t copy what others do irrespective of the circumstances. It is worth reiterating certain features of our experimental paradigm that we described in the introduction. On the one hand, and unlike previous studies which show that shared group membership impacts mimicry [21,39], participants don’t know who the other group members are. All they do know of any given dot is its colour which tells them if it is ingroup or outgroup. It is therefore possible to distinguish effects of shared group membership from effects of interpersonal relationships in our studies and to conclude that the former alone impacted mimicry.

On the other hand, and again in distinction from previous studies, other group members don’t know who the participant is. Participants therefore cannot communicate anything about themselves (such as their willingness to follow others and to act as a ‘good group member’) through the way they move their dot. Such communication about the self—and the motives associated with it (such as bonding with other group members and securing one’s acceptance by them)—are therefore unlikely as an explanation of our findings. However, it is possible that participants felt judged through the behaviour of their avatar. Future research could include post-study interviews to assess the extent to which participants believed they were anonymous and unobserved.

By contrast, our findings are compatible with a heuristic approach [28], albeit modified to state that the acts of other group members are diagnostic of how one should act oneself (as long as these are relevant to one’s group identity). In this way our findings concerning mimicry are in line with self-categorisation research on explicit social influence [47,48]. As such, the results allow us to conceptualize mimicry as more closely related in terms of underlying process to other social influence phenomena (not only conformity, but also minority influence, group polarization, and leadership [46]) than previously thought. Given that the study did not measure internalisation of beliefs or the time in which participants reacted, we cannot entirely rule out alternative processes of conformity or normative influence, although these seem unlikely given that the tasks involved the adoption of fleeting behaviours rather than attitude change. Future research could adapt the paradigm to test these alternative explanations. Either way, it is clear from our findings that group identification shaped social influence.

Nonetheless, the nature of our task does not rule out communicational process entirely, nor does it rule out affiliation motives but, critically, these would need to be conceptualised at a collective rather than an individual level. That is, while no-one knows that ‘my dot’ is ‘me’, they do know (by its colour) that it is an ingroup member. So, I can use the movement of the dot to demonstrate (for instance) that group members hang together and thereby seek to enhance the overall cohesion of the group.

Altogether, then, the studies reported in this paper provide evidence that we don’t mimic just anyone; rather mimicry is moderated by the mere fact of whether the source is seen as an ingroup member or an outgroup member and that this can occur independently of any personal relations we may have with that person.

In terms of underlying process, our studies rule out conventional explanations in terms of individual level communication and affiliation motives. They point instead to collective level explanations. However, whether these processes are epistemic (based on a heuristic that one should concur with fellow group members) or communicational/affiliation (based on the desire to make one’s group cohesive) or a mixture of both remains a topic for further investigation. Since that there was greater mimicry of ingroup than outgroup members, affiliation through appeasement [25,26] did not seem to be a factor in our study. This makes sense given that our stimuli were not threatening.

In terms of practical relevance, our findings have important implications for designing effective behavioural interventions. In contexts such as emergency evacuation, the egress behaviour of others is sometimes the only information people have about appropriate exits. Imitation has been observed in egress behaviour previously [29,30,64], but the self-categorization basis of such imitation has not been examined before. A self-categorization model of mimicry, in which people are more likely to follow ingroup than outgroup members, may be able to account for variations in copying behaviour in emergency contexts [32,33], and thus inform emergency preparedness planning and strategy in order to maximise public compliance with safety protocols. However, the external validity of the current research is potentially limited by its somewhat artificial nature. Fieldwork and simulation research would help to bridge the gap between the methods used here and practical interventions.

A further limitation of the current design is that due to the seating arrangements participants could have potentially observed each other’s devices and thus linked dot behaviour to individuals. Although we think that this possibility was unlikely, future versions of the paradigm could use dividers or have participants take part in different rooms to prevent any observation of one another’s behaviour in the tasks. This would also allow for the study of whether the physical co-presence of other participants had an influence on behaviour. However, given that the physical co-presence of participants was consistent across conditions in the current study, we do not believe that either of these alterations would affect the pattern of results.

In conclusion, the current study contributes to a better understanding of the influence of group membership on mimicry. People do not copy others in a way that is as inevitable and uncontrolled as is sometimes assumed. And if we understand the categorical conditions under which people do or don’t copy what others do, then we will see that even (perhaps especially) in the most extreme circumstances, copying behaviour can be harnessed as an asset rather than feared as a problem.

## Supporting information

S1 Table Parameter estimates for Bayesian mixed model of maze data. (DOCX)

S2 Table Estimates of condition contrasts for Bayesian mixed model of maze data. (DOCX)

S3 Table Results of mixed model analysis of maze data. (DOCX)

S4 Table Parameter estimates for Bayesian mixed model of rather vertical data. (DOCX)

S5 Table Estimates of condition contrasts for Bayesian mixed model of rather vertical data. (DOCX)

S6 Table Results of mixed model analysis of rather vertical data. (DOCX)

S7 Table Parameter estimates for Bayesian mixed model of rather horizontal data. (DOCX)

S8 Table Estimates of condition contrasts for Bayesian mixed model of rather horizontal data. (DOCX)

S9 Table Results of mixed model analysis of rather horizontal data. (DOCX)

S10 Table Parameter estimates for Bayesian mixed model of fidget data. (DOCX)

S11 Table Estimates of condition contrasts for Bayesian mixed model of fidget data. (DOCX)

S12 Table Results of mixed model analysis of fidget data. (DOCX)
