# Supplementary material for: Self-categorization as a basis of behavioural mimicry: Experiments in The Hive
Source: PLoS One. 2020 Oct 30;15(10):e0241227. doi: 10.1371/journal.pone.0241227 (PMC7598449; doi:10.1371/journal.pone.0241227)
Supplement: S1 Table — (DOCX) [file pone.0241227.s001.docx]

**Supporting Information**

The data analysed in this paper is publicly accessible here: <https://osf.io/kjt4u/?view_only=4a871bdea56a4f4e829c1bd651c5b97d>

We employed Bayesian Mixed Models for our analyses. Below we give a fuller description of the model parameter estimates, and estimates of condition contrasts, following Makowski (2018). This information was derived by fitting a Markov Chain Monte Carlo model. Weakly informative priors from the binomial family were used that were scaled by the rstanarm package. We used random effects for the experimental group, and fixed effects for the participants dot colour, the grouping method, the number of confederates and, when relevant, the orientation of the confederates. Random slopes and intercepts were used. We fitted a Markov Chain Monte Carlo binomial (link = logit) model (4 chains, each with iter = 2000; warmup = 1000; thin = 1; post-warmup = 1000).

**Maze model**

maze_side ~ colour * orientation + grouping + confederates +

(1 + colour * orientation + grouping + confederates | experimental group)

|  | Median | MAD | CI loW | CI high | MPE % |
| --- | --- | --- | --- | --- | --- |
| R2 | 0.3 | 0.02 | 0.27 | 0.33 |  |
| (Intercept) | 0.45 | 0.17 | 0.19 | 0.74 |  |
| Colour:red | -1.92 | 0.21 | -2.29 | -1.56 | 100.0 |
| Orientation:B | -1.62 | 0.22 | -1.97 | -1.28 | 100.0 |
| Grouping:TIPI | 0.21 | 0.17 | -0.08 | 0.5 | 89.5 |
| Confederates:HIGH | -0.14 | 0.16 | -0.42 | 0.11 | 81.4 |
| Colour x Orientation | 5.01 | 0.36 | 4.45 | 5.62 | 100.0 |

**Table 1. Parameter estimates for Bayesian mixed model of maze data**
