# Supplementary material for: Self-categorization as a basis of behavioural mimicry: Experiments in The Hive
Source: PLoS One. 2020 Oct 30;15(10):e0241227. doi: 10.1371/journal.pone.0241227 (PMC7598449; doi:10.1371/journal.pone.0241227)
Supplement: S2 Table — (DOCX) [file pone.0241227.s002.docx]

|  | Median | CI loW | CI high | MPE |
| --- | --- | --- | --- | --- |
| Colour | -0.66 | -.97 | -.38 | 100.0 |
| Orientation | -0.89 | -1.18 | -0.60 | 100.0 |
| Grouping | -0.29 | -0.58 | -0.01 | 94.4 |
| Confederates | 0.17 | -0.11 | 0.45 | 83.1 |

**Table 2. Estimates of condition contrasts for Bayesian mixed model of maze data**
