# Supplementary material for: Self-categorization as a basis of behavioural mimicry: Experiments in The Hive
Source: PLoS One. 2020 Oct 30;15(10):e0241227. doi: 10.1371/journal.pone.0241227 (PMC7598449; doi:10.1371/journal.pone.0241227)
Supplement: S3 Table — (DOCX) [file pone.0241227.s003.docx]

Priors used:

Intercept (after predictors centered)

~ normal(location = 0, scale = 10)

Coefficients

~ normal(location = [0,0,0,...], scale = [2.5,2.5,2.5,...])

Covariance

~ decov(reg. = 1, conc. = 1, shape = 1, scale = 1)

In addition, we performed a mixed model analysis using random intercepts only, as the model did not converge with random slopes. The model was specified as below, fit by REML, and t-tests used Satterthwaite's method

maze_side ~ colour * orientation + grouping + confederates + (1 | experimental group)

| Effect | df | F | p.value |
| --- | --- | --- | --- |
| Colour | 1,774.75 | 13.15 | .0003 |
| Orientation | 1,136.08 | 28.33 | <.0001 |
| Grouping | 1,152.90 | 1.65 | .20 |
| Confederates | 1,132.81 | 0.76 | .38 |
| Colour:Orientation | 1,774.47 | 345.92 | <.0001 |

**Table 3. Results of mixed model analysis of maze data**
