# Supplementary material for: Self-categorization as a basis of behavioural mimicry: Experiments in The Hive
Source: PLoS One. 2020 Oct 30;15(10):e0241227. doi: 10.1371/journal.pone.0241227 (PMC7598449; doi:10.1371/journal.pone.0241227)
Supplement: S4 Table — (DOCX) [file pone.0241227.s004.docx]

**Rather task– vertical positions model**

vertical dot position ~ colour * orientation + grouping + confederates +

(1 + colour * orientation + grouping + confederates | experimental group) +

(1 + colour * orientation + grouping + confederates | item)

|  | Median | MAD | CI loW | CI high | MPE % |
| --- | --- | --- | --- | --- | --- |
| R2 | 0.17 | 0.01 | 0.15 | 0.2 |  |
| (Intercept) | -0.15 | 0.02 | -0.17 | -0.11 |  |
| Colour:red | -0.02 | 0.02 | -0.05 | 0.01 | 90.2 |
| Confederates:HIGH | -0.01 | 0.02 | -0.05 | 0.03 | 72.3 |
| Grouping:TIPI | -0.01 | 0.02 | -0.04 | 0.02 | 76.9 |
| Orientation:B | -0.01 | 0.02 | -0.05 | 0.03 | 60.9 |
| Colour x Orientation | 0.04 | 0.02 | -0.01 | 0.09 | 92.8 |

**Table 4 Parameter estimates for Bayesian mixed model of rather vertical data**
