# Supplementary material for: Self-categorization as a basis of behavioural mimicry: Experiments in The Hive
Source: PLoS One. 2020 Oct 30;15(10):e0241227. doi: 10.1371/journal.pone.0241227 (PMC7598449; doi:10.1371/journal.pone.0241227)
Supplement: S5 Table — (DOCX) [file pone.0241227.s005.docx]

|  | Median | CI loW | CI high | MPE |
| --- | --- | --- | --- | --- |
| Colour | 0.002 | -0.02 | 0.02 | 57.9 |
| Orientation | -0.01 | -0.04 | 0.01 | 79.4 |
| Grouping | 0.01 | -0.02 | 0.04 | 73.4 |
| Confederates | 0.01 | -0.01 | 0.04 | 77.0 |

**Table 5. Estimates of condition contrasts for Bayesian mixed model of rather vertical data**
