# Supplementary material for: Self-categorization as a basis of behavioural mimicry: Experiments in The Hive
Source: PLoS One. 2020 Oct 30;15(10):e0241227. doi: 10.1371/journal.pone.0241227 (PMC7598449; doi:10.1371/journal.pone.0241227)
Supplement: S6 Table — (DOCX) [file pone.0241227.s006.docx]

Priors used:

Intercept (after predictors centered)

~ normal(location = 0, scale = 10) ** adjusted scale = 2.39

Coefficients

~ normal(location = [0,0,0,...], scale = [2.5,2.5,2.5,...])

**adjusted scale = [0.60,0.60,0.60,...]

Auxiliary (sigma)

~ exponential(rate = 1)

**adjusted scale = 0.24 (adjusted rate = 1/adjusted scale)

Covariance

~ decov(reg. = 1, conc. = 1, shape = 1, scale = 1)

In addition, we performed a mixed model analysis using random intercepts only, as the model did not converge with random slopes. The model was specified as below, fit by REML, and t-tests used Satterthwaite's method

vertical dot position ~ colour * orientation + grouping + confederates +

(1 + colour + orientation | experimental group) +

(1+ colour + orientation | item)

| Effect | df | F | p.value |
| --- | --- | --- | --- |
| Colour | 1, 61.44 | 0.58 | .45 |
| Grouping | 1, 141.21 | 0.12 | .73 |
| confederates | 1, 137.89 | 0.66 | .42 |
| orientation | 1,30.18 | 1.03 | .32 |
| Colour:orientation | 1,150.75 | 2.74 | .10 |

**Table 6. Results of mixed model analysis of rather vertical data**
