# Supplementary material for: Self-categorization as a basis of behavioural mimicry: Experiments in The Hive
Source: PLoS One. 2020 Oct 30;15(10):e0241227. doi: 10.1371/journal.pone.0241227 (PMC7598449; doi:10.1371/journal.pone.0241227)
Supplement: S7 Table — (DOCX) [file pone.0241227.s007.docx]

**Rather task– horizontal positions model**

horizontal dot position ~ colour * orientation + grouping + confederates +

(1 + colour * orientation + grouping + confederates | experimental group) +

(1 + colour * orientation + grouping + confederates | item)

|  | Median | MAD | CI loW | CI high | MPE % |
| --- | --- | --- | --- | --- | --- |
| R2 | 0.2 | 0.01 | 0.18 | 0.23 |  |
| (Intercept) | 0.08 | 0.14 | -0.24 | 0.42 |  |
| Colour:red | 0 | 0.05 | -0.14 | 0.14 | 52.9 |
| Confederates:high | -0.03 | 0.05 | -0.16 | 0.12 | 73.5 |
| Grouping:tipi | 0 | 0.12 | -0.34 | 0.33 | 51.3 |
| Orientation:B | -0.13 | 0.23 | -0.64 | 0.34 | 70.7 |
| Colour:Orientation | 0 | 0.12 | -0.33 | 0.39 | 50.6 |

**Table 7. Parameter estimates for Bayesian mixed model of rather horizontal data**
