# Supplementary material for: Self-categorization as a basis of behavioural mimicry: Experiments in The Hive
Source: PLoS One. 2020 Oct 30;15(10):e0241227. doi: 10.1371/journal.pone.0241227 (PMC7598449; doi:10.1371/journal.pone.0241227)
Supplement: S8 Table — (DOCX) [file pone.0241227.s008.docx]

|  | Median | CI loW | CI high | MPE |
| --- | --- | --- | --- | --- |
| Colour | 0.007 | -0.14 | 0.16 | 54.0 |
| Orientation | 0.12 | -0.26 | 0.54 | 68.0 |
| Grouping | 0.03 | -0.08 | 0.13 | 73.4 |
| Confederates | -0.003 | -0.24 | 0.25 | 50.1 |

**Table 8. Estimates of condition contrasts for Bayesian mixed model of rather horizontal data**
