# Supplementary material for: Self-categorization as a basis of behavioural mimicry: Experiments in The Hive
Source: PLoS One. 2020 Oct 30;15(10):e0241227. doi: 10.1371/journal.pone.0241227 (PMC7598449; doi:10.1371/journal.pone.0241227)
Supplement: S9 Table — (DOCX) [file pone.0241227.s009.docx]

Priors used:

Intercept (after predictors centered)

~ normal(location = 0, scale = 10) ** adjusted scale = 7.40

Coefficients

~ normal(location = [0,0,0,...], scale = [2.5,2.5,2.5,...])

** adjusted scale = [1.85,1.85,1.85,...]

Auxiliary (sigma)

~ exponential(rate = 1)

**adjusted scale = 0.74 (adjusted rate = 1/adjusted scale)

Covariance

~ decov(reg. = 1, conc. = 1, shape = 1, scale = 1)

In addition, we performed a mixed model analysis using random intercepts only, as the model did not converge with random slopes. The model was specified as below, fit by REML, and t-tests used Satterthwaite's method

horizontal dot position ~ colour * orientation + grouping + confederates +

(1 + colour + orientation | experimental group) +

(1+ colour + orientation | item)

| Effect | df | F | p.value |
| --- | --- | --- | --- |
| Colour | 1, 134.51 | 0.02 | .88 |
| Grouping | 1, 292.14 | 2.84 | .09 |
| confederates | 1, 266.70 | 0.26 | .61 |
| orientation | 1,3.00 | 0.13 | .74 |
| Colour:orientation | 1,419.65 | 0.04 | .85 |

**Table 9. Results of mixed model analysis of rather horizontal data**
