# Supplementary material for: Self-categorization as a basis of behavioural mimicry: Experiments in The Hive
Source: PLoS One. 2020 Oct 30;15(10):e0241227. doi: 10.1371/journal.pone.0241227 (PMC7598449; doi:10.1371/journal.pone.0241227)
Supplement: S10 Table — (DOCX) [file pone.0241227.s010.docx]

**Fidget model**

Total distance travelled ~ colour + grouping + confederates +

(1 + colour + grouping + confederates | experimental group)

|  | Median | MAD | CI loW | CI high | MPE % |
| --- | --- | --- | --- | --- | --- |
| R2 | 0.10 | 0.03 | 0.05 | 0.15 |  |
| (Intercept) | 0.89 | 0.04 | 0.83 | 0.97 |  |
| Colour:red | 0.09 | 0.04 | 0.02 | 0.16 | 98.0 |
| Grouping:tipi | 0.23 | 0.05 | 0.14 | 0.31 | 100.0 |
| Confederates:high | 0.03 | 0.05 | -0.05 | 0.11 | 73.7 |

**Table 10. Parameter estimates for Bayesian mixed model of fidget data**
