# Supplementary material for: Self-categorization as a basis of behavioural mimicry: Experiments in The Hive
Source: PLoS One. 2020 Oct 30;15(10):e0241227. doi: 10.1371/journal.pone.0241227 (PMC7598449; doi:10.1371/journal.pone.0241227)
Supplement: S11 Table — (DOCX) [file pone.0241227.s011.docx]

|  | Median | CI loW | CI high | MPE |
| --- | --- | --- | --- | --- |
| Colour | -0.09 | -0.15 | -0.02 | 98.35 |
| Grouping | -0.23 | -0.31 | -0.15 | 100.0 |
| Confederates | -0.02 | -0.10 | 0.05 | 70.8 |

**Table 11. Estimates of condition contrasts for Bayesian mixed model of fidget data**
