# Supplementary material for: Self-categorization as a basis of behavioural mimicry: Experiments in The Hive
Source: PLoS One. 2020 Oct 30;15(10):e0241227. doi: 10.1371/journal.pone.0241227 (PMC7598449; doi:10.1371/journal.pone.0241227)
Supplement: S12 Table — (DOCX) [file pone.0241227.s012.docx]

Priors used:

Intercept (after predictors centered)

~ normal(location = 0, scale = 10) **adjusted scale = 6.18

Coefficients

~ normal(location = [0,0,0], scale = [2.5,2.5,2.5]) **adjusted scale = [1.54,1.54,1.54]

Auxiliary (sigma)

~ exponential(rate = 1) **adjusted scale = 0.62 (adjusted rate = 1/adjusted scale)

Covariance

~ decov(reg. = 1, conc. = 1, shape = 1, scale = 1)

In addition, we performed a mixed model analysis using random intercepts only, as the model did not converge with random slopes. The model was specified as below, fit by REML, and t-tests used Satterthwaite's method.

Total distance travelled ~ colour + grouping + confederates +

(1 + colour + grouping + confederates | experimental group)

| Effect | df | F | p.value |
| --- | --- | --- | --- |
| Colour | 1, 135.01 | 4.34 | .04 |
| Grouping | 1, 96.07 | 14.79 | .0002 |
| confederates | 1, 119.04 | 0.91 | .34 |

**Table 12. Results of mixed model analysis of fidget data**
